# Life Cycle Assessment of Phycocyanin Food Colorant Production from Spirulina (*Arthrospira platensis*) with Biostimulant Waste-Stream Utilization for Soil Carbon Sequestration to Achieve Net Carbon Removal

**DOI:** 10.3390/foods15040610

**Published:** 2026-02-07

**Authors:** Asger Smidt-Jensen, Trine Boje Røgild, Tomer Cohen, Shahar Meshoulam, Lihie Iuclea, Hafþór Ægir Sigurjónsson, Asaf Tzachor, Margrét Geirsdóttir, William R. Moomaw

**Affiliations:** 1 Department of Food Science, Aarhus University, Agro Food Park 48, 8200 Aarhus, Denmark; 2 Centre for Food Technology, Danish Technological Institute, Kongsvang Allé 29, 8000 Aarhus, Denmark; 3 Harvard Kennedy School, Harvard University, 79 John F. Kennedy Street, Cambridge, MA 02138, USA; 4 Amot-BDO Bdlg, 48 Menachem Begin Rd., Tel Aviv 6618001, Israel; 5 KPMG ehf, Borgartúni 27, 105 Reykjavik, Iceland; 6 Matís, Vínlandsleið 12, 113 Reykjavik, Iceland; 7 Institute for Technology and Humanity, CSER, University of Cambridge, Level 1, 16 Mill Lane, Cambridge CB2 1SB, UK; 8 Center for International Environment & Resource Policy, Tufts University, 160 Packard Ave., Medford, MA 02155, USA

**Keywords:** Spirulina, life cycle assessment, soil organic carbon, photobioreactors, carbon negativity, carbon sequestration

## Abstract

This study introduces a novel approach to producing carbon-negative food ingredients by integrating phycocyanin extraction from Spirulina (*Arthrospira platensis*) with the application of its residual biomass as a biostimulant for soil organic carbon (SOC) sequestration. A comprehensive life cycle assessment (LCA) was conducted to evaluate the environmental performance of this integrated system, encompassing geothermally powered Spirulina cultivation, phycocyanin extraction, and the use of the waste stream to enhance SOC in degraded Icelandic soils. Although the cultivation and extraction processes are associated with environmental impacts, the SOC sequestration resulting from biostimulant application more than offsets these burdens—yielding a net-carbon-negative natural food colorant under the assumptions applied in this study (−1.60 tCO_2_-eq per color unit). This work highlights the potential for such ingredients to contribute meaningfully to Scope 3 emission reductions, in line with science-based targets and the GHG Protocol. Traditionally, food pigments have been overlooked in carbon accounting due to their low inclusion rates and perceived minimal contribution to overall product footprints. This study reframes natural colorants as strategic levers for climate action, offering a pathway for food manufacturers to advance decarbonization while transitioning toward more sustainable, bio-based ingredients.

## 1. Introduction

The escalating severity of global climate change underscores the urgent need for scalable, cost-effective, and practical carbon-negative solutions—particularly in the food production sector, which contributes approximately 30% of total anthropogenic greenhouse gas (GHG) emissions [1]. Within this sector, the majority of emissions are embedded in complex global supply chains, especially during the agricultural production phase, and land-use changes associated with raw material sourcing. From a corporate standpoint, these are classified as Scope 3 emissions. These indirect emissions occur across the value chain. For food and beverage manufacturers and retailers, Scope 3 emissions can account for as much as 95% of their total carbon footprint, presenting both a major decarbonization challenge and an opportunity for targeted intervention [2]. Reducing these emissions is therefore a critical priority for aligning corporate climate strategies with science-based targets and international reporting standards [3,4].

Researchers and policymakers have recently focused significantly on reducing emissions, particularly emphasizing methods that actively remove carbon dioxide (CO_2_) from the atmosphere [5], focusing on several major GHG-emitting sectors. Currently, more than 7000 businesses across regions and industries have adopted emission reduction targets aligned with climate science requirements through science-based targets (SBTs) [3]. Research by CDP [6] and WRI [7] shows that within the food and beverage sector, supply chain emissions (Scope 3) typically exceed direct operational emissions (Scope 1 and 2) by a factor of three to five. Despite some initial industry initiatives targeting supply chain decarbonization, widespread implementation remains sporadic and insufficient.

In the broader transition toward carbon-negative and climate-positive strategies, a range of pathways are being actively explored. These include agroforestry, regenerative land management, and the integration of innovative biological processes that couple food production with atmospheric CO_2_ removal [8]. Within this landscape, LCA-based carbon-negative ingredients offer a promising avenue for the food industry to advance its net-zero targets across both product and corporate levels.

At the product level, carbon-neutral or carbon-zero claims often rely on external offsets or credit purchases—an approach increasingly subject to criticism regarding transparency and impact [9]. In contrast, the use of ingredients with verifiable carbon-negative LCAs constitutes a legitimate and traceable means of reducing a product’s overall carbon footprint, in line with recognized GHG accounting protocols. By incorporating such ingredients, companies can credibly support carbon-neutral product claims without relying solely on external offset mechanisms.

At the corporate level, the procurement of carbon-negative ingredients becomes part of the company’s value chain emissions profile—particularly within Scope 3 categories covering supplier (upstream) and distribution (downstream) activities. Because Scope 3 typically represents the largest share of emissions for food manufacturers and retailers, integrating carbon-negative inputs provides a strategic lever for reducing reported GHG emissions and progressing toward corporate net-zero commitments in a science-aligned manner.

One category of ingredients that holds particular promise for advancing both sustainability and market differentiation is natural food colorants—an expanding segment with growing relevance for climate-smart innovation.

Industry analysis projects the food colorants sector will expand from USD 3.78 billion in 2024 to USD 10.40 billion by 2033, representing an 11.9% compound annual growth rate [10]. The current shift from artificial color additives to natural colorants is propelled by both regulatory and consumer demand requirements. For example, artificial colors are facing strict scrutiny due to potential health concerns arising from their use, as well as consumer demand for “cleaner” labels [11].

To meet growing demand for natural colorants while advancing sustainability goals, microalgae have emerged as a promising biological platform, offering both high pigment yield and favorable environmental characteristics.

Microalgae, a group encompassing diverse photosynthetic microorganisms such as cyanobacteria (e.g., *Arthrospira platensis*, Spirulina) and green algae (e.g., Chlorella) thrive in various aquatic environments, freshwater, brackish water, and highly saline habitats, and have an extraordinary ability to reproduce under the right conditions [12]. The simpler cellular structures of microalgae compared to more advanced plants allow for efficient energy use and rapid growth, allowing them to improve their ability to absorb nutrients and CO_2_. Microalgae could be a viable source for vibrant pigments, such as FAO-approved, phycocyanin-rich Spirulina (*Arthrospira platensis*) extract for industrial food coloring usage [13,14]. Phycocyanin, as part of the phycobiliprotein family, is a well-known antioxidant that is used as a natural blue colorant in confectionery products such as gum, panned chocolate and various candy applications [15]. As a primary color, blue can also be used to create other colors such as purple and green, potentially extending the carbon-negative footprint to a wider range of color applications. The common industrial metric used for phycocyanin color value is “color units (CUs) per kg.” This characteristic is commonly used by food manufacturers to assess the strength of Spirulina extract for usage as food colorant, without measurement of or conversion to phycocyanin content [16].

This value is calculated by measuring the absorbance of a buffered solution at 618 nm (A618nm):

CU kg^−1^ = (A618nm) × 10 × DF × (g × 100)^−1^

where: DF is the Dilution Factor and g is the sample weight in grams.

Against this background, this paper introduces a novel concept: a life cycle assessment (LCA)-based, carbon-negative food ingredient, achieved by valorizing the biostimulant-rich waste stream generated during phycocyanin extraction from Spirulina. The carbon-negative value of the ingredient derives primarily from its application to degraded soils, where it enhances soil organic carbon (SOC) sequestration—a key carbon drawdown mechanism.

While life cycle assessments have increasingly been applied to food products and production systems, ingredients with low inclusion rates (such as natural colorants) are often overlooked in carbon accounting due to their perceived marginal contribution to overall product footprints. To date, little attention has been given to the potential for such ingredients to contribute meaningfully to Scope 3 emission reductions when assessed on an appropriate functional-unit basis.

To quantify the environmental performance of this approach, a system-wide life cycle assessment (LCA) was conducted, encompassing three major stages: algal biomass cultivation, downstream processing for phycocyanin extraction, and end-use application of the residual biomass.

The biomass source is GeoSpirulina, a Spirulina cultivation system powered by geothermal energy and previously characterized in a recent study [17]. This cultivation provides both the target product—phycocyanin, a natural blue food colorant—and the process waste stream. Emissions associated with downstream extraction are calculated based on a published LCA of phycocyanin production [18]. Separately, a detailed LCA was performed for the end-of-life phase, focusing on the application of the biostimulant waste stream to Icelandic soils via precision agricultural drones [19]. This analysis includes geospatial adjustments to reflect Iceland’s average SOC levels, required application areas, and context-specific operational factors.

In addition to the environmental assessment, this study presents a use-case scenario that evaluates the implications of adopting the carbon-negative colorant at both the product level (e.g., in confectionery manufacturing) and the corporate level (with implications for Scope 3 emission accounting). The paper discusses the benefits, feasibility, and limitations of this approach, and proposes a broader portfolio of carbon-negative ingredients as a strategic avenue for food system decarbonization. This integrated pathway enables food manufacturers to address sustainability goals while simultaneously meeting consumer demand for natural and more responsible products.

### 1.1. Production of Colorants from Microalgae

Two primary approaches characterize microalgae production: open and enclosed cultivation methods. Open raceway configurations utilize mechanical circulation to maintain culture movement [12]. These ponds are considered relatively inexpensive to build and easy to expand if needed, making them popular for mass and modular cultivation of species such as Spirulina [20]. However, these systems bring challenges such as exposure and contamination by other organisms, as well as limited temperature control, and are considered unsustainable due to their extensive evaporative water loss [21].

Enclosed photobioreactor systems (PBRs) address these limitations through enhanced environmental control, enabling precise management of illumination, carbon dioxide levels, nutrient delivery, and thermal conditions [12]. PBRs are typically made of transparent tubes or flat plates, allowing optimal light distribution for even algae exposure. Concentrated CO_2_ can be added directly into the vessel at the desired optimum level, accelerating biomass growth. Advanced designs include artificial lighting (e.g., LEDs) tuned to microalgae’s specific photosynthetic irradiance (PAR) spectrum. Although more expensive, photobioreactors can achieve substantially higher biomass densities than open ponds, reducing the land footprint required for algae production [22].

Though one kilogram of microalgal biomass stoichiometrically captures about 1.8 kg of CO_2_, the carbon footprint associated with PBR cultivation, due to the energy consumption and nutrient demand, typically results in an unsustainable algal biomass carbon footprint. A recent study on European photobioreactor operations found that conventional microalgae production generates approximately 68 kg CO_2_-eq kg^−1^ [23]. Nevertheless, a recent LCA study employed a cradle-to-gate LCA of large-scale Spirulina (GeoSpirulina, *Arthrospira platensis*) cultivation in geothermally powered, LED-based PBRs in Iceland [17]. This system utilized geothermal infrastructure for renewable electricity, thermal regulation systems, process water, and carbon dioxide supply. The assessment revealed emissions of −8.0 kg CO_2_-eq per tonne of Spirulina biomass produced mainly due to biofixation—a virtually net-zero biomass production.

Commercial-scale extraction of pigments from algae, mainly phycocyanin, usually begins with dense cultures of the Spirulina algae. Once harvested, via a rotating sieve, the algal biomass is dried. Then, the cells undergo mechanical or enzymatic disruption to release their intracellular contents. The liquid fraction is clarified, and the pigment fraction can be further purified by filtration [22]. The resulting product is a vibrant blue powder or solution suitable for coloring various food products, from confectionery and dairy products (e.g., ice cream) to beverages (e.g., smoothies).

Phycocyanin production generates two primary waste streams. The first is a non-soluble fibrous mass that is rich in proteins and lipids, commonly referred to as residual biomass. This material holds potential for nutrient recycling applications [24,25]. The second is a water-soluble stream containing a dilute mixture of amino acids, peptides, and carbohydrates. Notably, some of the amino acids present in this fraction are recognized as plant biostimulants [26]. However, in most solar-based Spirulina production systems, the composition of this waste stream fluctuates on both daily and seasonal scales [27], making its consistent use as a biostimulant impractical. In contrast, GeoSpirulina production maintains a year-round consistent composition [28], resulting in a stable waste-stream profile suitable for more predictable applications.

### 1.2. Plant Biostimulants

Plant biostimulants are formulations that enhance vegetative development and auxiliary physiological functions of plants, including nutrient utilization efficiency, stress resilience, yield characteristics, and soil nutrient bioavailability [29]. Microbial biostimulant variants primarily include mycorrhizal fungi and beneficial rhizosphere bacteria [30]. Non-microbial categories encompass six primary types: chitosan, humic and fulvic acid, protein hydrolysates from animal or plant sources, phosphite compounds, algal extracts, and silicon. Additionally, plant-derived extracts represent an emerging biostimulant category receiving considerable recent attention.

A recent comprehensive analysis of 180 field studies globally demonstrated that biostimulant application produces an average of 17.9% of yield enhancement with corresponding photosynthetic carbon capture increases and soil application methods showing optimal efficacy. Despite minimal application rates (typically a few grams to tens of grams per hectare), these materials produce catalytic effects. Performance gains were most pronounced in degraded soils with depleted organic matter and nutrient deficiencies [26,31]. Similarly, a study on carrot root systems documented a 17% increase in sequestered organic carbon following combined soil and leaf amino acid biostimulant treatments. The usage of biostimulants for soil organic carbon (SOC) sequestration is a well-accepted carbon dioxide removal (CDR) methodology [26,32].

### 1.3. Soil Organic Carbon Sequestration

Within terrestrial carbon dynamics, soils constitute the predominant reservoir, functioning either as atmospheric CO_2_ sources or sinks [33,34]. Increasing soil carbon stocks represents a key strategy for atmospheric carbon dioxide mitigation in response to anthropogenic climate change [35]. Quantifying soil organic carbon involves sampling protocols that are affected by biological activity, soil density, mineral content, and depth profiles. Large-scale SOC assessment faces challenges from high spatial and temporal heterogeneity driven by variation in soil composition and climatic conditions as well as topographic and biological factors [36]. Developing reliable, efficient, and economically viable SOC quantification methods remains essential for implementing soil carbon management strategies at meaningful scales [34].

Iceland has experienced depletion of approximately 500 million tonnes of soil carbon reserves over eleven centuries of human habitation, subsequently released to the atmosphere [37]. Despite adequate rainfall across much of the nation, land degradation is an ongoing risk to natural resources. National soil assessments document severe erosion affecting 40% of Iceland’s land area [38,39]. This degradation presents a substantial opportunity for carbon restoration through ecosystem rehabilitation. An Icelandic study documented carbon accumulation in revegetated degraded landscapes across 62 sites in 32 regions, measuring annual organic carbon sequestration rates of 1.2 tonnes per hectare in both soil and plant biomass [40]. Projections suggest that this sequestration rate could persist for more than 100 years in restored desert areas.

## 2. Materials and Methods

**Figure 1:**
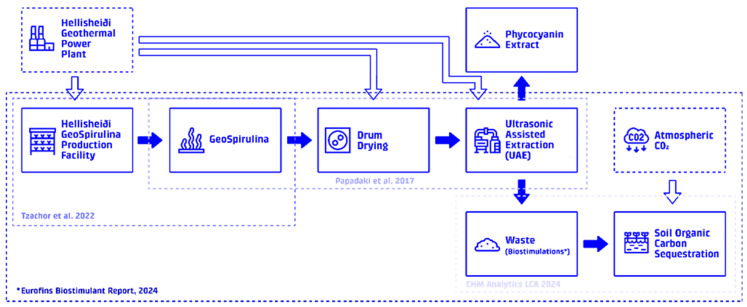
Process flow diagram: Flow chart illustrating the integrated process of Spirulina cultivation, phycocyanin extraction, and biostimulant application (highlighted by dashed boxes). Data is drawn from [17,18,19]. * The waste stream acts as a biostimulant when applied in a dilute water-based mixture, this process involves the use of agricultural drones.

This study investigates the potential of utilizing the waste fraction from phycocyanin blue food colorant production from *Arthrospira platensis* as a biostimulant for carbon sequestration in Icelandic soil. A process flow diagram (PFD) can be seen in Figure 1. The study employs a life cycle assessment (LCA) methodology, adhering to ISO 14040:2006 and ISO 14044:2006 standards [41,42], to evaluate the environmental impacts of the entire process, from Spirulina cultivation to food colorant extraction, biostimulant application, and subsequent carbon uptake. The LCA uses the attributional EF 3.1 methodology within the OpenLCA software (version 2.4.0). Water use and land use are also assessed as critical impact categories. Environmental burdens and benefits were allocated using a physical mass-based allocation approach, in accordance with ISO 14044.

### 2.1. Spirulina Cultivation and Phycocyanin Extraction

Spirulina cultivation data is derived from a study on large-scale Spirulina production at the Hellisheidi Geothermal Park in Iceland [17]. This facility utilizes closed photobioreactors (PBRs) powered by geothermal energy, receiving inputs such as CO_2_ for biofixation, freshwater, and macronutrients sourced from natural open mines. The cultivation process includes modified Zarrouk medium, controlled temperature (31 ± 2 °C), pH (10.8 ± 0.2), and a specific red/blue/UV illumination regime (3.5 W/L, 750 μmol/m^2^ s) using LEDs. Harvesting involves a continuous process where 15% of the culture is transferred to a rotation sieve for washing and downstream processing using micro- and ultrafiltration membranes. Residual medium water is treated and recycled.

Downstream processing for phycocyanin extraction follows methods previously outlined in the literature [18]. This involves the use of ultrasound-assisted extraction (UAE) with water as a solvent. The yield of this extraction is 3.22% of the dry biomass, extracted as phycocyanin. The specific extraction conditions, including biomass type (wet or dry), solvent (water), solid–liquid ratio, temperature, ultrasound frequency, and power, are selected based on achieving optimal phycocyanin yield while minimizing environmental impact. The remaining waste fraction (15.8% of the dry biomass), post-phycocyanin extraction, serves as the biostimulant. The technical documentation for this biostimulant-stream, including its composition and safety assessment, was performed by Eurofins Agroscience Services. This analysis confirmed the compliance and presence of lysine (3.92% wt.) and phenylalanine (3.22% wt.) as the primary amino acid components of this waste stream. It was found to be safe for usage and exempted from registration, as listed in Annex IV of REACH, with no regulatory classification (EC) 1272/2008 (CLP)/GHS.

It is important to note that this LCA focuses on the initial production of phycocyanin at the factory gate in Iceland and does not include further processing steps such as additional purification, re-formulation, packaging, or shipping that would be undertaken by color manufacturers before the product reaches food producers.

### 2.2. Biostimulant Application and Carbon Sequestration

Biostimulant application data is drawn from a prospective LCA of biostimulant application in Iceland [19]. This LCA focuses on the application of a Spirulina-derived biostimulant in Icelandic soil using agricultural drones. The biostimulant, initially in dry form, is mixed with water on-site before being loaded onto the drones. The LCA considers emissions associated with transporting equipment to the application site, drone operation (including energy consumption and component replacement), and monitoring of the treated area. The projected increase in soil organic carbon (SOC) is based on primary data undertaken by Matís ohf in Iceland. This waste handling process, while secondary to the primary objective of phycocyanin production, contributes to overall environmental performance by sequestering carbon. Based on the primary field study, described below, the annual application of 1 g DW of biostimulant can sequester approximately 25.0 kg of soil organic carbon per year. This translates to approximately 91.8 kg CO_2_-eq of carbon sequestration per g of biostimulant applied. In order to assure removal permanence through ongoing monitoring, this LCA uses a 50% reserve approach, where only half the removal is accounted for. Therefore, the biogenic removal numbers presented reflect a conservative conversion rate of 45.9 kg CO_2_-eq of carbon sequestration per g of biostimulant applied.

**Figure 2:**
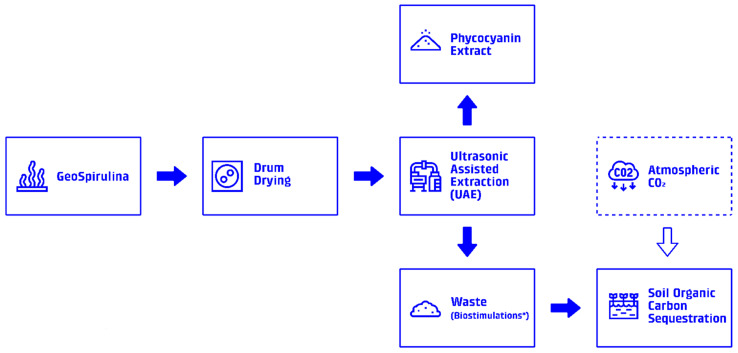
System boundary diagram: Visualization of the system boundaries for the LCA, showing all processes from Spirulina cultivation to biostimulant application and carbon sequestration. * The waste stream acts as a biostimulant when applied in a dilute water-based mixture, this process involves the use of agricultural drones.

The system boundary for the combined LCA encompasses all processes from Spirulina cultivation and phycocyanin extraction to biostimulant application and carbon uptake, as seen in Figure 2. The functional unit (FU) is defined as 1 color unit (CU) produced for utilization in the food industry at factory gate ready for distribution, equivalent to approximately 7.12 g of phycocyanin (approx. 140 CU kg^−1^). The selected system boundaries and operational parameters are consistent with established approaches [5]. A life cycle inventory for the entire system can be found in Table 1.

The carbon sequestration per g of biostimulant usage is calculated based on primary data of increased Icelandic soil organic stocks, as a result of Spirulina-waste-based biostimulant application. This was based on primary data sampled by Matís ohf which investigated changes in soil organic carbon (SOC) stocks in Iceland following the application of 47.6 g ha^−1^ yr^−1^ of GeoSpirulina-waste-based biostimulants. The field experiment was conducted in Ölfus, Iceland (64°04′18″ N, 21°28′59″ W) across two 12.45 ha sites: an experimental application site (EXP) and a nearby control site (CTRL). Spatial separation between the sites minimized potential biostimulant drift. The waste biostimulant was applied to EXP. An agricultural drone (DJI T25) applied the biostimulant solution (0.47% *w*/*w* in water) at a rate of 10.12 L ha^−1^, equivalent to 47.6 g ha^−1^.

Baseline soil samples (T_0_) were collected, followed by post-treatment sampling (T_1_), approximately a year later. Sampling was performed using a Wintex 2000 soil sampler (26 mm probe diameter) to a depth of 60 cm. At T_0_, nine samples were collected from EXP and six from CTRL. At T_1_, nine samples were collected from each site. Sampling protocols were consistent, executed by a 3rd party (VEL ehf., Reykjavík, Iceland), and GPS coordinates were recorded for each sampling point. A homogeneity test using 10 replicate measurements of a single soil sample yielded a coefficient of variation (CV) of 3.44%, confirming analytical precision and low variability. The applied soil sampling and SOC quantification approach follows established methodologies commonly used in field-based soil carbon assessments [37].

### 2.3. Life Cycle Impact Assessment and Interpretation

The environmental impacts of the entire process are evaluated using the EF 3.1 methodology implemented in OpenLCA software. This approach enables the characterization of multiple impact categories.

Climate change is measured as Global Warming Potential over 100 years (GWP100), expressed in kg CO_2_-equivalents, and includes both emissions from production and application processes as well as carbon sequestration in soils resulting from biostimulant use.

Water use is quantified using the Available WAter REmaining (AWARE) method, which incorporates regional water scarcity and accounts for the total freshwater consumed throughout the life cycle—including direct use in Spirulina cultivation, extraction, and biostimulant application, as well as indirect use associated with energy production and transportation. The results are reported in m^3^ world-equivalents, offering a regionally weighted impact assessment.

Land use is assessed via several indicators, including soil quality index, biotic production potential, erosion resistance, and mechanical filtration potential. This evaluation captures both the quantity of land occupied (m^2^·year) and qualitative changes in land condition. For Spirulina cultivation, the analysis considers the use of non-arable land within geothermal parks, while for biostimulant application, it examines the ecological effects on treated natural lands in Iceland.

The results are presented in terms of impact per functional unit (1 color unit delivered), allowing for a comprehensive evaluation of the net environmental benefits of utilizing the waste stream for carbon sequestration. The contribution of the waste handling process to the overall environmental performance is highlighted, emphasizing the carbon sequestration benefit.

The interpretation phase analyzes the relative contributions of different life cycle stages to each impact category. Particular attention is given to comparing the environmental burden of phycocyanin production with the environmental benefits of using its waste stream as a biostimulant. This includes quantifying the avoided impacts from conventional waste disposal methods and the positive impacts from carbon sequestration. The analysis also identifies potential trade-offs between impact categories, such as instances where increased water use might be justified by substantial carbon sequestration benefits.

### 2.4. Case Study: Application in Chocolate Dragee Confectionery

To investigate the potential environmental impact of utilizing phycocyanin as a blue colorant on a product level, a case study was made. The case study investigated the impact of using phycocyanin as a blue colorant in the confectionery industry, and a chocolate dragee product was selected due to it being a common use case for artificial blue colorants. Furthermore, the results of this case study were translated into what the use of a carbon-negative ingredient could mean at a corporate level for reaching net-zero goals. Here, we investigated how many CUs of phycocyanin are needed to compensate for a company’s Scope 1, 2, and 3 emissions. To do this, 4 major food production/ingredient companies’ ESG reports were investigated, and CO_2_ emissions from Scope 1, 2, and 3 were averaged. This was used to give an estimate of how many CUs of phycocyanin a company would need to achieve a 5%, 10%, 20% and 50% reduction in total carbon footprint.

The inventory data of the chocolate dragee product were established based on the product’s ingredients list and the calculations follow a cradle-to-gate perspective. The results are presented as impact per 100 g of mixed-color chocolate dragee product. The calculation includes raw materials, production processes and packaging.

The investigated chocolate dragee consisted of 67% milk chocolate, 27% sugar, 1% E414 gummi arabicum used as a stabilizer, 0.5% Tapioca starch, and 0.5% colorant. For packaging, a multilayer packaging consisting of 5 g polyethylene terephthalate (PET), 1 g low-density polyethylene (LDPE), and 4 g polypropylene (PP) was considered.

Chocolate dragees are produced by pouring liquid tempered chocolate between two freezing rolls, forming a web of chocolate pieces. After cooling, the web is broken to release the individual shapes, and any leftover crumbs of chocolate are melted and reused. The shaped chocolate is then coated with multiple layers of high-dry-matter sugar syrup (approx. 70° Brix) containing food colorant and subsequently dried in rotating pans or drums resembling cement mixers. The layers are dried while the tumbling motion smooths the surface. A final sealant or polish is often applied for a glossy finish [43].

The chocolate dragees are then filled into packaging and are ready to be shipped to customers. In this case study, it was not possible to obtain inventory data for production of chocolate dragees. Therefore, 30% of the carbon footprint from the raw materials is used as the assumed carbon footprint of the production phase. This will most likely result in an overestimation for the production-phase emissions. However, this is done due to a lack of data and to make a conservative assumption.

In the calculation, a bag of mixed-color chocolate dragee was considered, and different scenarios were taken into account. In the base case scenario, it was assumed that the bag contained 5 different colors, where each color of dragee accounted for 20% of the bag’s mass. The 5 colors considered were: red/violet (E163—Anthocyanins), yellow (E100—Curcumin), orange (E160a—Beta-caroten), caramel (E150a—Caramel color), and blue (E133—Synthetic Blue No 1).

In Scenario 2, the same conditions were considered. However, here the blue color of the chocolate dragees was assumed to be replaced by using phycocyanin as the colorant instead of E133. Here it was assumed that a value of 0.28 CU per kg of mixed-color chocolate dragees is needed to achieve a proper color of the product from phycocyanin. This dosage takes into account that only 1/5 of the dragees in the mixed bag would be colored blue. The inventory data can be found in Table 2.

### 2.5. Sensitivity Analysis

To evaluate the robustness of the LCA results and identify the parameters exerting the greatest influence on environmental impacts, a sensitivity analysis was carried out in accordance with ISO 14044 guidelines. This analysis examined how variations in key data and methodological assumptions affect the overall outcomes [44].

The process began with the selection of key parameters based on their relevance and associated uncertainties. These included (a) the carbon sequestration efficiency of the biostimulant, (b) energy consumption during cultivation and extraction, (c) biomass yield and phycocyanin content, and (d) transport distances for biostimulant application.

Each parameter was then varied individually by ±20%, while keeping all other parameters constant, to simulate a moderate uncertainty range. The resulting changes in climate change impact, measured as GWP100, were calculated and expressed as percentage deviations from the baseline result.

In addition to these one-at-a-time parameter variations, four scenario analyses were conducted to explore the influence of alternative system configurations. Scenario 1 assumed a conservative estimate of carbon sequestration, with a 50% reduction in sequestration rates. Scenario 2 modeled an optimized cultivation setup, achieving a 90% reduction in energy consumption. Scenario 3 explored the use of ground-based application methods instead of drone-based spraying. Scenario 4 assumed a high phycocyanin content in the biomass, increasing from 3.22% to 20%. Together, these analyses provide insight into the sensitivity of environmental outcomes to both operational variables and systemic changes.

## 3. Results

### 3.1. Environmental Impact of Spirulina Cultivation

The environmental impact assessment of Spirulina cultivation at the Hellisheidi Geothermal Park reveals a significantly lower carbon footprint compared to conventional microalgae production systems. Based on the underlying study, the production of 1 kg of Spirulina biomass in this facility results in −0.008 kg CO_2_-eq GHG emissions, effectively making it carbon-neutral. However, the more conservative approach adopted in this study using the same data results in 4.19 kg CO_2_-eq per kg of wet Spirulina biomass, primarily attributed to the higher average impact of electricity consumption used (average Icelandic data rather than from a specific plant). This impact covers only cultivation of 1 kg of wet Spirulina biomass and therefore does not include further processing. This results in a climate impact of 0.15 kg CO_2_-eq per CU delivered. Land-use and water-use impact are relatively low at 0.19 Pt and 0.057 m^3^ deprived, respectively.

### 3.2. Environmental Impact of Phycocyanin Extraction

The downstream processing for phycocyanin extraction contributes the most significant environmental burden in the production chain. The ultrasound-assisted extraction process requires energy inputs and generates waste streams. Based on the literature inventory data used, the extraction of phycocyanin to deliver 1 CU results in approximately 4.83 kg CO_2_-eq of GHG emissions, primarily attributed to the drying process. However, this impact is balanced by the carbon sequestration potential of the waste stream when used as a biostimulant.

### 3.3. Carbon Sequestration Potential of Biostimulant Application

The application of the waste biostimulant on Icelandic soils demonstrates significant potential for carbon sequestration. The change in SOC from the field trial in Iceland was statistically significant (Kruskal–Wallis test, *p* < 0.05). At the 90% confidence level, the lower bound of the estimated annual increase in SOC stocks was 1.34 tC ha^−1^ yr^−1^, corresponding to a relative annual increase of 4.4%. The applied annual biostimulant rate is consistent with recommended application ranges for commercial amino-acid-based biostimulant products (2.0–48.0 g ha^−1^ yr^−1^) [26].

The reported annual SOC increase is comparable to the average regenerative (non-biostimulant-based) SOC sequestration rate in Icelandic soils of 1.2 tC ha^−1^ yr^−1^ [40]. However, the observed relative increase of 4.4% is conservative compared with findings from a meta-analysis of biostimulant field applications on root and tuber crops (13 studies), which reported an average annual increase in SOC stocks of 10.6% [31].

Based on the LCA data and field studies, the application of 1 g of biostimulant (diluted) can sequester approximately 12.50 kg of SOC after applying a 50% reversal buffer. This translates to approximately 45.90 kg CO_2_-eq of carbon sequestration per g of biostimulant applied.

The total carbon sequestration potential from the waste stream of phycocyanin production significantly outweighs the emissions associated with cultivation and extraction. For every 1 CU of phycocyanin delivered, the corresponding waste, when applied as a biostimulant, can sequester approximately 1.60 tCO_2_-eq, resulting in a net-carbon-negative product.

### 3.4. Net Environmental Impact of the Integrated System

**Figure 3:**
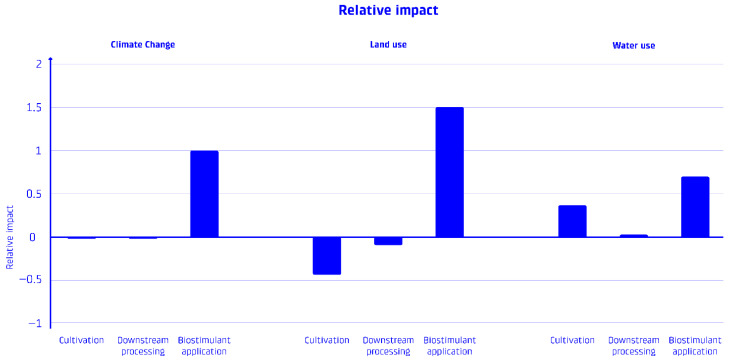
Environmental impact comparison: Comparison of the environmental impacts (climate change, land use, and water use) of the different production stages: cultivation, downstream processing (extraction, drying, packaging), and biostimulant application.

**Figure 4:**
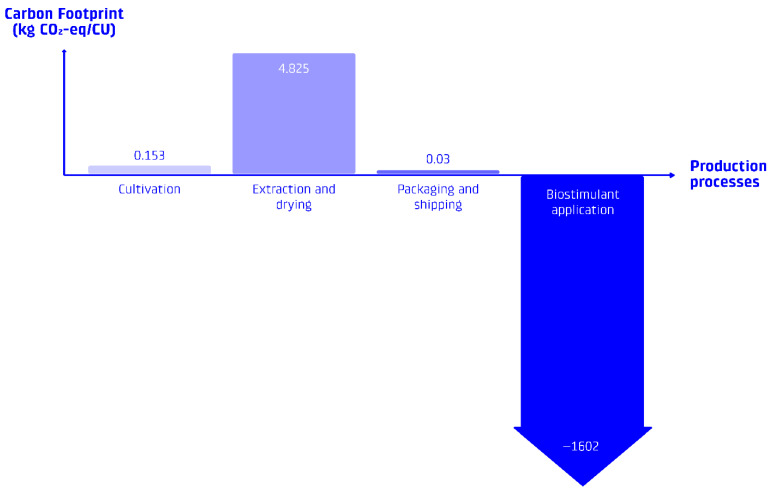
Carbon balance visualization: Proportion of carbon footprint by stage of production. Note that biostimulant application is not to scale.

The integrated system of Spirulina cultivation, phycocyanin extraction, and biostimulant application indicates a net-carbon-negative system; see Figure 3. The total GHG emissions from cultivation and extraction are more than balanced by the carbon sequestration achieved through biostimulant application, resulting in a net carbon footprint of approximately −1.60 tCO_2_-eq per CU of phycocyanin delivered; see Figure 4 for a visual overview of the process contributions. On top of the applied reversal buffer, to account for uncertainties regarding potential data variability, an additional conservative downsizing margin of 30% was applied in case study calculations (−1.12 tCO_2_-eq per CU of phycocyanin delivered).

Water use remains a significant environmental impact, primarily due to the cultivation phase. However, the water-use efficiency of the Hellisheidi facility, coupled with the substantial carbon sequestration benefits, presents a favorable trade-off in terms of overall environmental performance.

Land-use impact is minimal for cultivation but more significant for biostimulant application. However, the application targets degraded and desertified lands in Iceland, which benefit from the revegetation and soil improvement facilitated by the biostimulant, resulting in a net positive impact on land quality.

### 3.5. Case Study Results: Chocolate Dragee Confectionery

**Figure 5:**
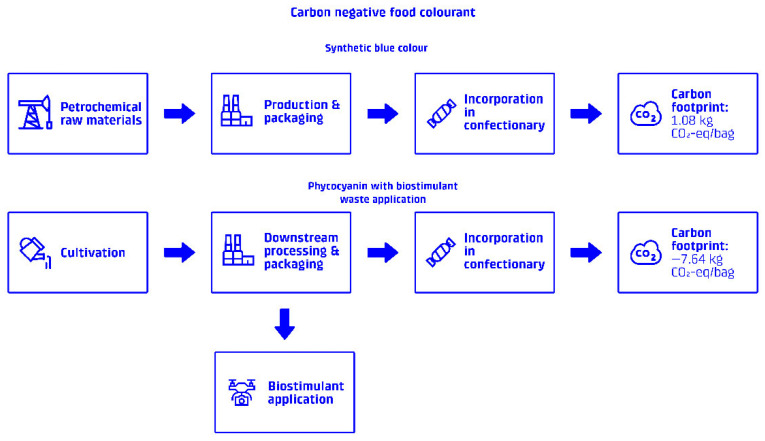
Case study application: Visual representation of the chocolate dragee confectionery case study, showing the incorporation of the carbon-negative blue colorant and its contribution to reducing the product’s overall carbon footprint.

This case study investigated the use of phycocyanin as a carbon-negative colorant in a 100 g bag of mixed-colored chocolate dragees, with blue accounting for 20% of the color—a common use case of artificial blue colorants. In the baseline scenario, the total carbon footprint of the product was calculated to be 1.08 kg CO_2_-eq per bag. By replacing the blue colorant (E133) with phycocyanin blue, which requires 0.28 CU per kg of chocolate dragees, the product’s carbon footprint was reduced to −7.64 kg CO_2_-eq per bag. This change represents a significant reduction due to the utilization of phycocyanin’s waste stream as a biostimulant. As mentioned above, in order to account for uncertainties, 30% of the emission reduction was removed from the calculations. The results can be seen in Figure 5.

At the corporate level, the use of phycocyanin was assessed as a potential strategy to contribute to net-zero emissions. To assess the potential, an average carbon footprint was calculated using Scope 1, 2, and 3 emissions from four major food/ingredient production companies. This amounted to a total carbon footprint of ~26.4 million tCO_2_-eq. Based on this, it was calculated how many bags of phycocyanin-colored chocolate dragees a tangible reduction of 5%, 10%, 20% and 50% would require. This was also converted to how many CUs of phycocyanin each reduction potential target requires. The results can be seen in Table 3.

While complete emissions balancing through a single ingredient is an unrealistic goal, this calculation helps illustrate the potential impact of carbon-negative ingredients in a company’s broader sustainability strategy. The daily production capacity of one of the largest producers of chocolate dragees is over 400 million individual dragees. This corresponds to over one billion bags per year. Thus, realistically, a single color replacement in a single product could help this corporation to achieve over 20% of its 2050 net-zero goals—a significant contribution to the company’s GHG management program.

The results demonstrate that phycocyanin has significant potential as a carbon-negative ingredient in the food industry. Its use as a colorant can not only reduce the climate impact at product level but also enable substantial CO_2_ reductions at the corporate level, contributing to net-zero goals. However, further research is needed to validate the long-term carbon sequestration effects and assess the cost and practical challenges of large-scale implementation.

### 3.6. Sensitivity Analysis and Uncertainty

**Figure 6:**
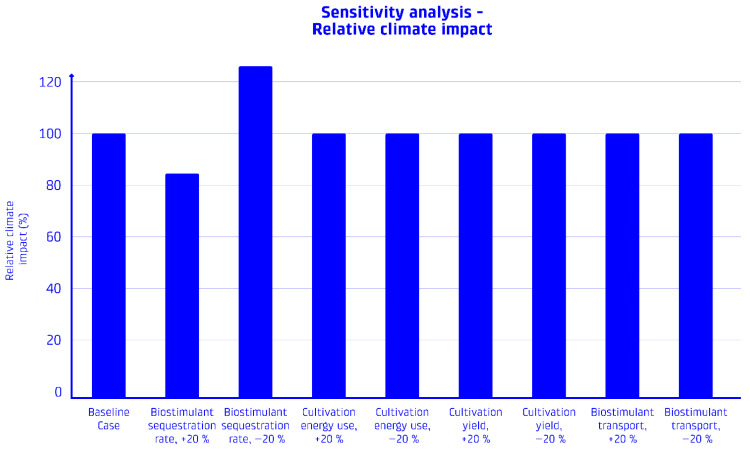
Sensitivity analysis results: Results of the sensitivity analysis of the net climate impact on key parameters such as biostimulant sequestration rate and cultivation yield.

The sensitivity analysis revealed that the environmental performance of the phycocyanin production system with biostimulant utilization is most sensitive to specific parameters related to carbon sequestration and production efficiency. Table 4 presents the results of the parameter sensitivity analysis for climate change impacts, while Figure 6 shows the relative climate impact.

The sensitivity analysis shows that the model results are most sensitive to changes in the carbon sequestration efficiency of the biostimulant. A 20% change in the sequestration rate leads to a corresponding ~20% change in the overall climate impact. This highlights the critical importance of accurate data on carbon sequestration rates in Icelandic soils.

In contrast, changes in energy consumption for cultivation, cultivation efficiency, and transport distances have minimal effects on the overall climate impact (less than 0.1% change). This is due to the dominance of the carbon sequestration benefit in the overall environmental profile.

Four alternative scenarios were evaluated to assess their impact on the overall climate performance; see Table 5.

The scenario analysis demonstrates that even under conservative carbon sequestration assumptions (Scenario 1), the system remains strongly carbon-negative. Improvements in cultivation efficiency (Scenario 2) and changes in application method (Scenario 3) have negligible effects on the overall climate impact due to the relatively small contribution of these processes to the total impact.

Most significantly, Scenario 4 examines the effect of a higher phycocyanin content in the algal biomass (20% versus the baseline 3.22%). This product-to-waste ratio change has profound implications for the carbon-negative potential of the system. With higher phycocyanin content, less waste biomass is generated per CU of phycocyanin produced, resulting in less biostimulant available for soil application and consequently reduced carbon sequestration per functional unit.

This causes an 83.9% reduction in the carbon-negative potential, though the system still maintains a substantial net negative carbon footprint (−257.21 kg CO_2_-eq CU^−1^).

This finding highlights that the carbon-negative potential is heavily dependent on the product-to-waste ratio. While higher phycocyanin content might be economically advantageous from a production efficiency perspective, it significantly reduces the climate benefit per functional unit. This creates an interesting sustainability trade-off between production efficiency and carbon sequestration potential that must be carefully considered in system optimization.

Moreover, several sources of uncertainty were identified in this assessment. First, the effectiveness of the biostimulant in enhancing carbon sequestration presents a notable source of uncertainty, as long-term empirical data under varied Icelandic soil conditions remain limited. Drawing on previous field trials, this parameter is estimated to have moderate uncertainty (±20%). Second, the permanence of sequestered carbon over the 100-year assessment horizon introduces additional uncertainty. To address this, conservative assumptions were applied regarding long-term carbon retention rates. Third, although energy consumption data are based on actual operations at the production facility, some variability in energy-use patterns leads to low uncertainty, estimated at ±10%. Lastly, the use of extraction data from the literature, which reflects laboratory-scale conditions, introduces further uncertainty when extrapolated to industrial-scale processes.

The sensitivity analysis highlights that the overall climate impact is most sensitive to assumptions regarding biostimulant-driven carbon sequestration. While conservative measures were applied (including a 50% reversal buffer and additional uncertainty deductions), the magnitude of sequestration remains subject to variability related to soil type, climatic conditions, and long-term management practices. Consequently, the reported net-carbon-negative results should be interpreted as conditional on the specific field data and assumptions applied in this assessment.

The identification of carbon sequestration efficiency as the key sensitivity parameter aligns with the contribution analysis results presented in Section 4.4, confirming that optimization of this aspect should be the primary focus for ensuring environmental benefits of the system.

## 4. Discussion

The results of this study demonstrate the substantial potential of creating carbon-negative food ingredients through the integration of phycocyanin production from Spirulina with the utilization of its waste stream as a biostimulant for SOC sequestration. A comprehensive LCA reveals that this integrated approach successfully transforms what would typically be considered a waste product into a valuable resource that contributes to climate change mitigation.

The intensive carbon-negative footprint achieved through this process (−1.12 tCO_2_-eq per CU of phycocyanin, after a 30% uncertainty deduction and 50% reversal buffer) represents a paradigm shift in the GHG impact of natural food colorants and how food ingredients can be produced and evaluated from an environmental perspective. While conventional food colorant production typically generates positive carbon emissions, this approach demonstrates that it is possible to create ingredients that actively remove carbon dioxide from the atmosphere, providing a double benefit of both replacing artificial colorants with natural alternatives and meaningfully contributing to climate change mitigation.

The sensitivity analysis demonstrates that the carbon-negative nature of the integrated phycocyanin production and biostimulant application system is robust across a wide range of parameter variations. Even under conservative assumptions about carbon sequestration efficiency (50% reduction), the system maintains a substantial carbon-negative profile. However, the analysis of varying phycocyanin content reveals a critical consideration for this carbon-negative approach. The product-to-waste ratio fundamentally determines the carbon sequestration potential per functional unit. With a phycocyanin content of 20% instead of 3.22%, the carbon-negative potential decreases by 83.9%, though the system remains carbon-negative. This creates an interesting sustainability paradox: while higher phycocyanin content might improve production efficiency and economic viability, it simultaneously reduces the climate benefit per unit of product. This relationship between product yield and environmental impact highlights the need for careful system optimization that balances economic, resource and environmental objectives.

The case study of chocolate dragees further illustrates the practical application of this approach, showing how incorporating carbon-negative phycocyanin as a colorant can transform a conventional confectionery product with a positive carbon footprint (1.08 kg CO_2_-eq per 100 g bag) into one with a substantially negative footprint (−7.64 kg CO_2_-eq per bag). This shift highlights the potential for carbon-negative ingredients to play a transformative role in helping food manufacturers reduce their Scope 3 emissions.

However, several important considerations must be addressed to fully realize the potential of this integrated system at scale. These include the challenges of allocating environmental benefits among multiple products, addressing scalability and implementation barriers, ensuring the permanence of carbon sequestration, and establishing the economic viability of the approach. While the results demonstrate strong carbon removal potential under Icelandic conditions, extrapolation to other regions should be undertaken cautiously and supported by site-specific data.

In addition, the practical implementation of carbon-negative ingredient claims raises important considerations related to regulatory acceptance, labeling standards, and industrial scalability. Carbon removal claims associated with soil sequestration are subject to increasing scrutiny with respect to permanence, verification, and attribution within product-level and corporate carbon accounting frameworks. These aspects may influence how carbon-negative ingredients can be communicated and adopted at scale, despite their mitigation potential. The following sections will explore these aspects in detail, providing a critical assessment of the opportunities and challenges associated with this innovative approach to more sustainable food ingredient production.

### 4.1. Carbon Accounting Implications

The carbon-negative approach to phycocyanin production presented here aligns robustly with established GHG accounting frameworks and can provide significant strategic value for corporate sustainability targets, particularly under the Science-Based Targets initiative (SBTi)’s Forest, Land, and Agriculture (FLAG) guidance. Central to this approach is the concept of regenerative production, wherein the process of cultivating Spirulina and extracting phycocyanin inherently contributes to regenerating degraded land through the application of the Spirulina biomass waste as a biostimulant. This approach distinctly positions phycocyanin as a regenerative raw material within the value chain of natural coloring products, allowing end-users (e.g., food and beverage companies) to leverage verified carbon-negative claims within their Scope 3 removals inventory.

The GHG Protocol Product Life Cycle Accounting Standard [45] supports the approach of attributing biogenic removals—specifically, soil organic carbon (SOC) sequestration—to the primary product if the co-product’s value and usage materially justify its inclusion within system boundaries. In this context, the significant SOC sequestration from applying Spirulina biomass waste as a biostimulant to degraded lands is a legitimate, quantifiable carbon removal recognized by the GHG Protocol’s Product Standard.

When compiling a corporate Scope 3 inventory, companies use the GHG Protocol’s Corporate Standard [4] and the Value Chain (Scope 3) Standard [46]. According to the Value Chain Standard, a product-specific life cycle analysis result is the most accurate method for calculating emissions from purchased goods and services (Category 1). Yet the Corporate and Value Chain Standards use a different approach to the Product Standard when it comes to carbon removal. According to these standards, inventories for Scope 1, Scope 2 and Scope 3 must represent gross emissions, accounting only for emissions and excluding any removals. Removals should be reported separately and not used to offset emissions. This inconsistency between the standards presents a challenge for companies purchasing carbon-negative products. Rather than using the final result of the LCA, companies need to identify the emissions and removals associated with the product and report them separately.

Because of the unique nature of FLAG emissions, the SBTi permits using a net emissions figure, which includes removals in FLAG targets. The biostimulant-driven SOC sequestration represents a nature-based solution that can contribute toward corporate net-zero FLAG targets. The FLAG framework explicitly recognizes regenerative agricultural practices, including SOC enhancement through soil amendments, as valid carbon removal mechanisms eligible for inclusion in corporate climate action strategies in FLAG sectors. Hence, phycocyanin, when produced under such regenerative practices, inherently supports companies with FLAG emission reduction targets.

The carbon accounting treatment for phycocyanin production places emphasis on Scope 3 removals driven by SOC sequestration, rather than on the relatively minor emissions arising from Scope 1 and 2 activities (e.g., electricity and drying processes). Specifically, the primary carbon-negative impact occurs through the downstream use of the Spirulina biomass waste as a biostimulant, substantially enhancing SOC when applied to degraded agricultural or barren lands.

From the perspective of the phycocyanin producer, these SOC sequestration benefits are associated with waste generated during production and thus qualify as Scope 3 removals (Category 1—Purchased Goods and Services) for the end-user buying the phycocyanin-containing product. Proper external verification and transparent reporting ensure the integrity and credibility of this removal claim, mitigating any risks of double-counting.

The requirements for reporting removals are detailed in the GHG Protocol Land Sector and Removals Guidance (2022 draft) [47]. Companies setting science-based targets for the FLAG sector are required to use this guidance for their reporting. Removal accounting needs to be kept separate from emission accounting. For companies purchasing natural coloring products containing regenerative phycocyanin, integration into corporate GHG inventories and climate disclosures involves accounting for carbon removals arising from SOC sequestration. Such end-users should explicitly report the carbon-negative impact as Scope 3 removals. The magnitude of this SOC sequestration warrants transparency and material disclosure within corporate sustainability reports, clearly differentiating this removal from conventional emission reduction strategies.

To credibly include carbon-negative inputs such as regenerative-produced phycocyanin within Scope 3 removals inventory and utilize these claims within SBTi-validated FLAG targets, end-users must adhere to stringent data quality requirements. First, a storage monitoring framework needs to be developed to ensure long-term storage and account for reversals if they occur through ongoing storage monitoring. Second, full traceability throughout the CO_2_ removal pathway needs to be established, including the sink to which carbon is transferred from the atmosphere and the pool where it is stored. Third, the reported net carbon stock changes need to be accounted for based on primary data from the specific sinks and pools where removal takes place. Fourth, when reporting removals, companies need to state the uncertainty range for the removal based on a specified confidence level, along with justification for how the stated value does not represent an overestimation. Finally, if a reversal occurs in a previously reported removal, companies need to either report this reversal as net carbon emissions for that reporting year or, if the carbon pools where the reversals occurred are no longer in the inventory boundary, report it as a reversal. In addition to the data quality requirements, in order to count removals in SBTi FLAG targets, companies must have in place a mechanism that ensures that there is no double-counting between companies. Adhering to these conditions is critical for ensuring the integrity and acceptance of removals reporting and SBTi-validated FLAG targets.

### 4.2. Permanence and Validation of Carbon Sequestration

As soil organic carbon sequestration is influenced by long-term land management, disturbance regimes, and environmental conditions, the permanence and reproducibility of sequestration outcomes remain a key source of uncertainty. For this reason, the carbon-negative balance reported in this study reflects a conservative, context-specific estimate rather than a universally transferable value.

Some methodologies for monitoring and validating SOC sequestration rely on soil samples. However, the soil samples can only give spot values and so methods were developed to interpolate the SOC values between the individual sample locations and create a carbon map of an area. Interpolation methods to produce the map, such as cubist, random forest and kriging [48], cannot be adapted to extrapolate beyond the sampled area to estimate the wider area levels of SOC. Improvements in interpolation methods have included geographically weighted regression [49].

The use of sampling in statistics normally involves a one-dimensional, finite population and the number of samples controls the uncertainty of the estimation of the wider population. The estimation of SOC using statistical methods allows for an infinite population. The estimation of uncertainty in soil sampling is two-dimensional and dependent not only on the number of samples [50] but also the samples’ locations. Also, if spatial autocorrelation (spatial variability) is not considered, the estimation of SOC may be very different than if the SOC is randomly distributed [51]. The pattern of the sampling is especially important if the dataset has high variability [52,53,54,55,56]. The sampling depth is also a factor that can influence the overall estimation of soil organic carbon stocks (SOCS) [57].

Validation methods should be designed to ensure the sample accuracy reflects the entire population under study. Many different methods of validating the sample data have been developed; common methods include cross-validation (k-fold [KFO], leave-one-out [LOO], leave-one-group-out [LOGO], and leave-one-field-out [LOFO]) [58].

The development of Artificial Intelligence (AI) has created the possibility of using machine learning for measuring and monitoring SOCS within and beyond sampled areas. SOC sample results and relevant predictors, such as multispectral imagery, have been used to train machine learning systems to interpolate and extrapolate SOCS [59,60]. Machine learning methods have a high upfront cost as dense sample data, covering a wide range of land use, is required for training.

Practices such as minimized cultivating, cover cropping, crop rotation, time-managed grazing and agroforestry can enhance soil health and increase SOC stability. These methods reduce soil disturbance, increase organic matter input, and promote microbial activity, all of which contribute to long-term carbon storage and protect against SOC loss. Adding organic materials such as crop residue, compost, and manure to soils can improve soil structure and biology to increase SOC content and protect previous gains. A complex set of processes stabilize organic carbon and prevent loss and are supported by improved biology and structure including microbial decomposition, chemical recalcitrance, physical protection and organo-mineral association. Developing policies that support sustainable land management and provide incentives for farmers to adopt carbon-friendly practices can play a significant role in ensuring SOC permanence. This includes carbon credits and subsidies for sustainable farming practices. By integrating these approaches, we can enhance the permanence of sequestered soil organic carbon and contribute to long-term climate mitigation efforts.

### 4.3. Challenges and Limitations

A significant challenge in assessing the environmental impact of the integrated system is the allocation of benefits among multiple potential products derived from Spirulina biomass. While this study focuses on phycocyanin as the primary product, Spirulina can yield various co-products, including vitamins [28], proteins, lipids, and other high-value compounds, as well as algal biomass-based biochar [61]. If multiple products are extracted from the same biomass, the carbon sequestration benefits from the waste stream would need to be allocated proportionally among these products.

The allocation method chosen (e.g., mass-based or economic-value-based) can significantly influence the calculated environmental impact of each product. For instance, if economic allocation is applied, a larger share of the carbon sequestration benefit might be attributed to higher-value products, potentially reducing the carbon-negative potential of phycocyanin.

While the findings highlight the promising potential of an integrated system for producing carbon-negative food colorants, scaling up this approach entails several challenges. First, significant infrastructure investments are required to expand Spirulina cultivation and phycocyanin extraction capacity. This includes the development of photobioreactors, specialized extraction equipment, and processing facilities.

Second, although Spirulina cultivation itself occupies minimal land, the biostimulant application component necessitates access to degraded or marginal lands suitable for carbon sequestration. The availability and accessibility of such land may pose constraints on scalability. However, recent tools developed by the UN Food and Agriculture Organization may assist in assessing the potential for soil organic carbon (SOC) enhancement and help guide rational deployment strategies.

Third, regulatory frameworks could present barriers to broader implementation. While the specific biostimulant compounds have been deemed safe and exempt from registration under current EU regulations, other biostimulants derived from microalgae waste streams may face regulatory scrutiny—particularly when applied to natural ecosystems or agricultural landscapes.

Lastly, the commercial success of carbon-negative food colorants ultimately hinges on market acceptance. Adoption will likely depend on consumers’ and buyers’ willingness to pay a premium for sustainably produced alternatives.

Furthermore, potential non-permanence and reversal risks for SOC sequestration could be caused by mismanagement and natural hazard risks, such as wildfires, volcanic eruptions and earthquakes. Based on current knowledge and experience [62], wildfire risk in Iceland is minimal, with no known recent wildfires, mainly thanks to the typical Icelandic vegetation cover and the relative distance of grasslands from human activity. Although it is impossible to predict exactly when volcanoes become active, the current volcanic activity in Reykjanes is not expected to cause volcanic activity in other parts of Iceland. Keeping a minimal distance of 5 km from known active volcanic zones would render SOC projects “low risk”. Earthquakes are common in the SW part of Iceland but are mostly small. Larger earthquakes do happen, but it is hard to see how these would affect soil organic carbon permanence in sequestration projects. Therefore, biostimulant-based SOC sequestration projects in Iceland could be classified as having a “low risk of underperformance and reversals”.

Coupling SOC sequestration with agricultural activity might also present a reversal risk [63]. From this perspective, most of the lands in Iceland are not exposed to intensive agricultural activities (e.g., grassland, moss land, lava field, wetland, etc.). Icelandic soils are classified, based on the EUNIS classification system, using a range of field measurements, such as: percent cover of all plants; total vascular plant, moss, and lichen cover; cover of individual vascular plant species; vegetation height; soil depth; and soil carbon and pH [64,65]. Taking into account the high-SOC potential of degraded Icelandic soils, located at low-risk areas, about 37% of the Icelandic soils qualify for SOC sequestration application [40].

One of the key parameters to enable mass adoption of Scope 3 GHG emission reduction is cost-related. A recent CarbonGap analysis projected that companies would be willing to reduce ~1% of their profitability in order to achieve carbon neutrality [66]. In other words, they would be willing to pay, per 1 tonne of CO_2_-eq emission reduction, the equivalent of 1% of their profit per tonne of CO_2_-eq emission. Preliminary estimates show that the proposed solution of a carbon-negative food coloring ingredient is in line with the industry’s financial comfort zone [67].

### 4.4. Future Research Directions

The findings of this study point to several avenues for future research. First, further investigation into the composition and formulation of the biostimulant could enhance its carbon sequestration efficacy and enable broader application across diverse soil types and ecological contexts.

Second, long-term field trials are essential to validate the sustained carbon sequestration potential and assess the permanence of stored carbon under varying environmental conditions.

Third, exploring alternative uses for the waste stream—beyond its current application as a biostimulant—may uncover additional pathways to amplify the environmental benefits of Spirulina cultivation and processing.

Finally, research into other potential carbon-negative ingredients derived from microalgae could contribute to an expanded portfolio of sustainable inputs for the food industry, reinforcing the role of microalgae as a versatile and climate-smart resource.

## 5. Conclusions

This study demonstrates the substantial potential of integrating phycocyanin production from Spirulina with the utilization of its waste stream as a biostimulant for soil organic carbon (SOC) sequestration to create a carbon-negative food colorant.

Primary data from a field study suggest that biostimulant application can significantly increase soil organic carbon stocks in Icelandic soils under controlled conditions. A comprehensive LCA reveals that the carbon sequestration achieved through biostimulant application in degraded Icelandic soils (−1.60 tCO_2_-eq per CU) significantly exceeds the environmental impacts of Spirulina cultivation and phycocyanin extraction (4.98 kg CO_2_-eq per CU combined), resulting in a net carbon removal of −1.60 tCO_2_-eq per CU of phycocyanin delivered. A more conservative estimate, including a 30% reduction, results in −1.12 tCO_2_-eq per CU of phycocyanin. However, this carbon-negative potential is highly dependent on the product-to-waste ratio, with higher phycocyanin content significantly reducing the amount of waste stream available for carbon sequestration.

The case study of chocolate dragees provides compelling evidence of the practical application of this approach. By replacing 20% of the conventional colorants with carbon-negative phycocyanin, a standard confectionery product can be transformed from having a positive carbon footprint (1.08 kg CO_2_-eq per bag) to a negative one (−7.64 kg CO_2_-eq per bag), even when applying conservative estimates that account for uncertainties in long-term carbon sequestration.

At a corporate level, our analysis indicates that this approach could offer a viable pathway for food manufacturers to address their Scope 3 emissions, which typically constitute most of their total carbon footprint. The relatively low reversal risks of SOC sequestration in Iceland, coupled with the consistent composition of GeoSpirulina waste streams, provides a reliable foundation for this carbon-negative approach.

However, realizing this potential at scale will require addressing several challenges, including the allocation of environmental benefits among multiple products, infrastructure requirements for expanded production, regulatory considerations, and validation of long-term carbon sequestration.

This concept represents a promising pathway for the food industry to progress toward decarbonization goals while simultaneously transitioning to natural food ingredients. By advancing the development of carbon-negative food ingredients, this study contributes to the broader goal of developing practical, scalable, and cost-effective solutions for mitigating climate change while meeting growing consumer demand for more sustainable and natural food products, provided that uncertainty and context dependence are appropriately addressed.

Further research should focus on optimizing biostimulant formulations, conducting extended field trials to validate long-term carbon sequestration, exploring alternative applications for microalgae waste streams, and expanding the portfolio of potential carbon-negative ingredients derived from microalgae.

## Figures and Tables

**Table 1:** Inventory data for producing 1 color unit (CU) of phycocyanin and the subsequent application of its waste stream as a biostimulant for soil carbon sequestration. All values are normalized to the functional unit of 1 CU of biomass delivered at factory gate. For cultivation, extraction and biostimulant application, literature data sources were used [17,18,19].

Input	Amount	Unit	Data Source	Comments
**Aluminum, cast alloy [GLO]**	0.00434	kg	EcoInvent 3.9	For bioreactor production
**Battery, Li-ion [GLO]**	0.00011	kg	EcoInvent 3.9	For drone production
**Cable [GLO]**	1.83251 × 10^−5^	kg	EcoInvent 3.9	For drone production
**CO_2_**	0.40179	kg	Elementary flow	For cultivation
**CO_2_**	1.60248	t	Elementary flow	Carbon sequestration
**Diesel [GLO]**	8.13974	MJ	EcoInvent 3.9	For drone operation
**Electricity, high voltage [IS]**	1.76167	MJ	EcoInvent 3.9	Used in multiple processes
**Electricity, medium voltage [IS]**	118.38543	MJ	EcoInvent 3.9	Used in multiple processes
**Electronics, for control units [RER]**	0.00641	kg	EcoInvent 3.9	Used in multiple processes
**Glass-fiber-reinforced plastic, polyamide [RER]**	0.00594	kg	EcoInvent 3.9	Used in multiple processes
**Iron sulfate [GLO]**	2.23214 × 10^−5^	kg	EcoInvent 3.9	Nutrients
**Land occupation**	0.00320	m^2^ × a	Elementary flow	For cultivation
**Light-Emitting Diode (LED) [GLO]**	0.00538	kg	EcoInvent 3.9	For bioreactor production
**Nitrogen fertilizer, as N [GLO]**	0.01116	kg	EcoInvent 3.9	Nutrients
**Nylon 6-6 [RoW]**	2.19901 × 10^−6^	kg	EcoInvent 3.9	For drone production
**Polyethylene, high density [GLO]**	0.00011	kg	EcoInvent 3.9	Used in multiple processes
**Polypropylene [GLO]**	0.00014	kg	EcoInvent 3.9	For bioreactor production
**Polyvinylchloride [GLO]**	0.00141	kg	EcoInvent 3.9	For bioreactor production
**Silicone, at plant [GLO]**	0.00012	kg	EcoInvent 3.9	For bioreactor production
**Sodium phosphate [RoW]**	0.00893	kg	EcoInvent 3.9	Nutrients
**Steel, chromium steel 18/8 [GLO]**	0.00242	kg	EcoInvent 3.9	For bioreactor production
**Steel, unalloyed [RER]**	0.00155	kg	EcoInvent 3.9	For bioreactor production
**Tap water [IS]**	37.29687	kg	EcoInvent 3.9	Used in multiple processes
**Transport, freight, lorry 16–32 ton [GLO]**	6.96354 × 10^−5^	t × km	EcoInvent 3.9	Used in multiple processes
**Transport, freight, lorry 3.5–7.5 ton [GLO]**	1.75961	t × km	EcoInvent 3.9	Used in multiple processes
**Transport, freight, transoceanic ship [GLO]**	0.00443	t × km	EcoInvent 3.9	Used in multiple processes

**Table 2:** Inventory for chocolate dragee production.

Input	Amount	Unit	Data Source	Comments
**Milk chocolate, at plant [RER]**	0.067	kg	Agribalyse v.3.11	Used in multiple processes
**Sugar, from sugarcane [GLO]**	0.027	kg	Ecoinvent 3.11	Used in multiple processes
**Corn glucose syrup, at plant [GLO]**	0.004	kg	Agribalyse v.3.11	Used in yellow dragee
**Turmeric [FR]**	0.0001	kg	Agribalyse v.3.11	Used in yellow dragee. Proxy for Curcumin
**Potato starch [GLO]**	0.0015	kg	Ecoinvent 3.11	Used in multiple procesess. Proxy for stabilizer E414 gummi arabicum and Tapioca starch
**Spirulina (*Spirulina* sp.), dried [FR]**	0.0001	kg	Agribalyse v.3.11	Used in blue/green dragee. Proxy for E133—Synthetic Blue No 1
**Sugarcane processing, traditional annexed plant [RoW]**	0.0001	kg	Ecoinvent 3.11	Used as proxy for production of E150a
**Boiling, industrial, 1 kg of boiled product, for cooking [IS]**	0.0001	kg	Ecoinvent 3.11	Used as proxy for production of E150a. Adapted to islandic conditions
**Beetroot juice, pure juice [FR]**	0.0001	kg	Agribalyse v.3.11	Used in red/violet dragee. Proxy for E163—Anthocyanins
**Carrot juice, pure juice [FR]**	0.0001	kg	Agribalyse v.3.11	Used in orange dragee. Proxy for E160a Beta-caroten
**Dragee production**			Best estimate	30% of raw material carbon footprint
**Polyethylene terephthalate, granulate, bottle grade [GLO]**	0.005	kg	Ecoinvent 3.11	Used in packaging
**Polypropylene, granulate [GLO]**	0.004	kg	Ecoinvent 3.11	Used in packaging
**Extrusion, plastic film [GLO]**	0.01	kg	Ecoinvent 3.11	Used in packaging
**Polyethylene, low density, granulate [GLO]**	0.001	kg	Ecoinvent 3.11	Used in packaging

**Table 3:** Results for the use of phycocyanin as a potential strategy to contribute to carbon footprint emission reductions at a corporate level. Here reduction goals of 5%, 10%, 20% and 50% are assessed and related to how many bags of chocolate dragee or what amount of phycocyanin is needed to achieve the reduction goal.

Reduction Goal [%]	Emission Reduction [tCO_2_e.]	Bags of 100 g Mixed-Color Chocolate Dragee Product	Amount of Phycocyanin [CU]
**5**	1,318,724	246,541,553	138,063
**10**	2,637,448	493,083,105	276,127
**20**	5,274,897	986,166,210	552,253
**50**	13,187,242	2,465,415,526	1,380,633

**Table 4:** Sensitivity analysis results for key parameters in the phycocyanin production system.

Parameter	Change	Climate Impact (kg CO_2_-eq CU^−1^)	% Change from Baseline
**Baseline case**	-	−1597.50	0%
**Carbon sequestration efficiency**			
**Biostimulant sequestration rate**	+20%	−1917.95	−20.06%
**Biostimulant sequestration rate**	−20%	−1277.01	+20.06%
**Energy consumption**			
**Cultivation energy use**	+20%	−1596.47	+0.06%
**Cultivation energy use**	−20%	−1598.53	−0.06%
**Biomass yield**			
**Cultivation yield**	+20%	−1598.62	−0.07%
**Cultivation yield**	−20%	−1595.60	+0.12%
**Transport distances**			
**Biostimulant transport**	+20%	−1597.35	+0.01%
**Biostimulant transport**	−20%	−1597.65	−0.01%

**Table 5:** Results of scenario analysis for phycocyanin production.

Scenario	Climate Impact (kg CO_2_-eq CU^−1^)	% Change from Baseline
**Baseline**	−1597.50	0%
**Scenario 1: Conservative sequestration**	−793.96	+50.3%
**Scenario 2: Optimized cultivation**	−1603.89	−0.4%
**Scenario 3: Alternative application**	−1599.10	−0.1%
**Scenario 4: High phycocyanin content (20%)**	−257.21	+83.9%

## Data Availability

The original contributions presented in this study are included in the article. Further inquiries can be directed to the corresponding author.

## Abbreviations

The following abbreviations are used in this manuscript:

CO_2_-eq	Carbon dioxide equivalents
CU	Color unit
EF	Environmental footprint
FLAG	Forest, Land, and Agriculture
FU	Functional unit
GHG	Greenhouse gas
GWP100	Global Warming Potential (100-year time horizon)
LCA	Life cycle assessment
LCI	Life cycle inventory
PBR	Photobioreactor
SBTi	Science-Based Targets initiative
SOC	Soil organic carbon
SOCS	Soil organic carbon stocks

## References

[1] European Comission (2023). Field to Fork: Global Food Miles Generate Nearly 20% of All CO_2_ Emissions from Food. https://environment.ec.europa.eu/news/field-fork-global-food-miles-generate-nearly-20-all-co2-emissions-food-2023-01-25_en.

[2] Deloitte (2025). Towards Consistent Measurement of Scope 3. https://www.deloitte.com/uk/en/Industries/consumer/blogs/2023/towards-consistent-measurement-of-scope-3.html.

[3] (2025). Science Based Targets Initiative. https://sciencebasedtargets.org/.

[4] World Resources Institute, World Business Council for Sustainable Development (2013). GHG Protocol.

[5] Center for Climate and Energy Solutions (C2ES) (2025). Carbon Dioxide Removal. https://www.c2es.org/content/carbon-dioxide-removal/.

[6] CDP (2025). CDP Technical Note: Relevance of Scope 3 Categories by Sector.

[7] World Resources Institute (2022). Trends Show Companies Are Ready for Scope 3 Reporting with US Climate Disclosure Rule. https://www.wri.org/update/trends-show-companies-are-ready-scope-3-reporting-us-climate-disclosure-rule.

[8] Smith P., Clark H., Dong H., Elsiddig E., Haberl H., Harper R., House J., Jafari M., Masera O., Mbow C. (2014). Chapter 11—Agriculture, forestry and other land use (AFOLU). Climate Change 2014: Mitigation of Climate Change; IPCC Working Group III Contribution to AR5.

[9] Nguyen A. (2023). Carbon Neutral Claims Under Investigation in Greenwashing Probe. https://www.forbes.com/sites/amynguyen/2023/06/16/carbon-neutral-claims-under-investigation-in-greenwashing-probe/.

[10] Sharma A. (2025). Food Colors Market Size, Share & Trends Analysis Report.

[11] Caporgno M.P., Mathys A. (2018). Trends in Microalgae Incorporation into Innovative Food Products with Potential Health Benefits. Front. Nutr..

[12] Khan M.I., Shin J.H., Kim J.D. (2018). The promising future of microalgae: Current status, challenges, and optimization of a sustainable and renewable industry for biofuels, feed, and other products. Microb. Cell Factories.

[13] FDA (2023). Listing of Color Additives Exempt From Certification.

[14] FAO, WHO (2023). 95th JECFA—Chemical and Technical Assessment (CTA), 2022.

[15] Liu R., Qin S., Li W. (2022). Phycocyanin: Anti-inflammatory effect and mechanism. Biomed. Pharmacother..

[16] FAO, WHO (2018). Residue Monograph Prepared by the Meeting of the Joint FAO/WHO Expert Committee on Food Additives (JECFA), 86th Meeting 2018.

[17] Tzachor A., Smidt-Jensen A., Ramel A., Geirsdóttir M. (2022). Environmental Impacts of Large-Scale Spirulina (*Arthrospira platensis*) Production in Hellisheidi Geothermal Park Iceland: Life Cycle Assessment. Mar. Biotechnol..

[18] Papadaki S., Kyriokopoulou K., Tzovenis I., Krokida M. (2017). Environmental impact of phycocyanin recovery from *Spirulina platensis* cyanobacterium. Innov. Food Sci. Emerg. Technol..

[19] Hakkarainen V. (2024). Prospective LCA of Biostimulant Application in Iceland.

[20] Pereira H.G.C. (2019). Biotechnological Applications of a Promising Marine Chlorophyte (Tetraselmis SP. CTP4): A Biorefinery Approach.

[21] Moomaw W., Berzin I., Tzachor A. (2017). Cutting Out the Middle Fish: Marine Microalgae as the Next Sustainable Omega-3 Fatty Acids and Protein Source. Ind. Biotechnol..

[22] Gonçalves A.L. (2021). The Use of Microalgae and Cyanobacteria in the Improvement of Agricultural Practices: A Review on Their Biofertilising, Biostimulating and Biopesticide Roles. Appl. Sci..

[23] Mata T.M., Cameira M., Marques F., Santos E., Badenes S., Costa L., Vieira V.V., Caetano N.S., Martins A.A. (2018). Carbon footprint of microalgae production in photobioreactor. Energy Procedia.

[24] Sandia National Laboratories (2015). Algae nutrient recycling is a triple win. ScienceDaily.

[25] Barbera E., Bertucco A., Kumar S. (2018). Nutrients recovery and recycling in algae processing for biofuels production. Renew. Sustain. Energy Rev..

[26] Rosa R., Hajko L., Franczuk J., Zaniewicz-Bajkowska A., Andrejiová A., Mezeyová I. (2023). Effect of L-Tryptophan and L-Glutamic Acid on Carrot Yield and Its Quality. Agronomy.

[27] Kumar K.S., Kumari S., Singh K., Kushwaha P. (2021). Influence of Seasonal Variation on Chemical Composition and Nutritional Profiles of Macro- and Microalgae. Recent Advances in Micro and Macroalgal Processing: Food and Health Perspectives.

[28] Tzachor A., Oever S.P.V.D., Mayer H.K., Asfur M., Smidt-Jensen A., Geirsdóttir M., Jensen S., Smárason B.O. (2024). Photonic management of Spirulina (*Arthrospira platensis*) in scalable photobioreactors to achieve biologically active unopposed vitamin B12. Discov. Food.

[29] Jardin P.D. (2015). Plant biostimulants: Definition, concept, main categories and regulation. Sci. Hortic..

[30] Rouphael Y., Colla G. (2020). Editorial: Biostimulants in Agriculture. Front. Plant Sci..

[31] Li J., Gerreway T.V., Geelen D. (2022). A Meta-Analysis of Biostimulant Yield Effectiveness in Field Trials. Front. Plant Sci..

[32] Popko M., Michalak I., Wilk R., Gramza M., Chojnacka K., Górecki H. (2018). Effect of the New Plant Growth Biostimulants Based on Amino Acids on Yield and Grain Quality of Winter Wheat. Molecules.

[33] Scharlemann J.P., Tanner E.V., Hiederer R., Kapos V. (2014). Global soil carbon: Understanding and managing the largest terrestrial carbon pool. Carbon Manag..

[34] Wang S., Guan K., Zhang C., Lee D., Margenot A.J., Ge Y., Peng J., Zhou W., Zhou Q., Huang Y. (2022). Using soil library hyperspectral reflectance and machine learning to predict soil organic carbon: Assessing potential of airborne and spaceborne optical soil sensing. Remote Sens. Environ..

[35] Lal R. (2004). Soil carbon sequestration to mitigate climate change. Geoderma.

[36] Angelopoulou T., Tziolas N., Balafoutis A., Zalidis G., Bochtis D. (2019). Remote Sensing Techniques for Soil Organic Carbon Estimation: A Review. Remote Sens..

[37] Arnalds A. (2004). Carbon Sequestration and the Restoration of Land Health. Clim. Change.

[38] Arnalds A. (2000). Evolution of rangeland conservation strategies. Rangeland Desertification, Advances in Vegetation Science.

[39] Arnalds O., Thorarinsdottir E.F., Metusalemsson S., Jonsson A., Gretarsson E., Arnason A. (2001). Soil Erosion in Iceland.

[40] Arnalds O., Aradottir A.L., Gudbergsson G. (2002). Organic Carbon Sequestration by Restoration of Severely Degraded Areas in Iceland. Agricultural Practices and Policies for Carbon Sequestration in Soil.

[41] (2006). Environmental Management—Life Cycle Assessment—Principles and Framework.

[42] (2006). Environmental Management—Life Cycle Assessment—Requirements and Guidelines.

[43] Beckett S.T. (2008). Industrial Chocolate Manufacture and Use.

[44] Wei W., Larrey-Lassalle P., Faure T., Dumoulin N., Roux P., Mathias J.-D. (2014). How to Conduct a Proper Sensitivity Analysis in Life Cycle Assessment: Taking into Account Correlations within LCI Data and Interactions within the LCA Calculation Model. Environ. Sci. Technol..

[45] World Resources Institute, World Business Council for Sustainable Development (2011). Product Life Cycle Accounting and Reporting Standard.

[46] World Resources Institute, World Business Council for Sustainable Development (2011). Corporate Value Chain (Scope 3) Accounting and Reporting Standard.

[47] World Resources Institute, World Business Council for Sustainable Development (2022). Land Sector and Removals Guidance (Draft for Pilot Testing and Review).

[48] Pouladi N., Møller A.B., Tabatabai S., Greve M.H. (2019). Mapping soil organic matter contents at field level with Cubist, Random Forest and kriging. Geoderma.

[49] Song X.-D., Brus D.J., Liu F., Li D.-C., Zhao Y.-G., Yang J.-L., Zhang G.-L. (2016). Mapping soil organic carbon content by geographically weighted regression: A case study in the Heihe River Basin, China. Geoderma.

[50] Long J., Liu Y., Xing S., Qui L., Huang Q., Zhou B., Shen J., Zhang L. (2018). Effects of sampling density on interpolation accuracy for farmland soil organic matter concentration in a large region of complex topography. Ecol. Indic..

[51] Radočaj D., Jug I., Vukadinović V., Jurišić M., Gašparović M. (2021). The Effect of Soil Sampling Density and Spatial Autocorrelation on Interpolation Accuracy of Chemical Soil Properties in Arable Cropland. Agronomy.

[52] Booman G., Leiker S. (2021). Soil Sampling Guide.

[53] Ferguson R.B., Hergert G.W. (2009). Soil Sampling for Precision Agriculture. Precis. Agric..

[54] Guo M. (2009). Soil Sampling and Methods of Analysis. J. Environ. Qual..

[55] Loughran R.J., Wallbrink P.J., Walling D.E., Appleby P.G. (2003). Sampling Methods. Handbook for the Assessment of Soil Erosion and Sedimentation Using Environmental Radionuclides.

[56] Nehab D., Shilane P. Stratified Point Sampling of 3D Models. Proceedings of the Eurographics Symposium on Point-Based Graphics.

[57] Franzen D., Cihacek L. (1998). Soil Sampling as a Basis for Fertilizer Application.

[58] Stevens A., Miralles I., Wesemael B.V. (2012). Soil Organic Carbon Predictions by Airborne Imaging Spectroscopy: Comparing Cross-Validation and Validation. Soil Sci. Soc. Am. J..

[59] Castaldi F., Chabrillat S., Jones A., Vreys K., Bomans B., Wesemael B.V. (2018). Soil Organic Carbon Estimation in Croplands by Hyperspectral Remote APEX Data Using the LUCAS Topsoil Database. Remote Sens..

[60] Castaldi F., Hueni A., Chabrillat S., Ward K., Buttafuoco G., Bomans B., Vreys K., Brell M., Wesemael B.V. (2019). Evaluating the capability of the Sentinel 2 data for soil organic carbon prediction in croplands. ISPRS J. Photogramm. Remote Sens..

[61] Myung E., Kim H., Choi N., Cho K. (2024). The biochar derived from *Spirulina platensis* for the adsorption of Pb and Zn and enhancing the soil physicochemical properties. Chemosphere.

[62] Thorsteinsson P.T. (2025). Personal Communication.

[63] Oldfield E., Eagle A., Rubin R., Rudek J., Sanderman J., Gordon D. (2021). Agricultural Soil Carbon Credits: Making Sense of Protocols for Carbon Sequestration and Net Greenhouse Gas Removals.

[64] Natural Science Institute of Iceland (2021). Terrestrial Habitat Types. https://www.natt.is/en/flora-funga/habitat-types/terrestrial-habitat-types.

[65] European Environment Agency (EEA) (2022). EUNIS, the European Nature Information System. https://eunis.eea.europa.eu/index.jsp.

[66] Höglund R., Mitchell-Larson E. (2022). Bridging the Ambition Gap: A Framework for Scaling Corporate Funds for Carbon Removal and Wider Climate Action.

[67] Haflidasson K. (2025). Personal Communication.

